# Risk of thrombosis with thrombocytopenia syndrome after COVID‐19 vaccination prior to the recognition of vaccine‐induced thrombocytopenia and thrombosis: A self‐controlled case series study in England

**DOI:** 10.1002/rth2.12698

**Published:** 2022-04-20

**Authors:** Hannah Higgins, Nick Andrews, Julia Stowe, Gayatri Amirthalingam, Mary Ramsay, Gurpreet Bahra, Anthony Hackett, Karen A. Breen, Michael Desborough, Dalia Khan, Heather Leary, Connor Sweeney, Elizabeth Hutchinson, Susan E. Shapiro, Charlotte Lees, Jay Dhanapal, Peter K. MacCallum, Shoshana Burke, Vickie McDonald, Ngai Mun Aiman Entwistle, Stephen Booth, Christina J. Atchison, Beverley J. Hunt

**Affiliations:** ^1^ 371011 Health Protection Division UK Health Security Agency London UK; ^2^ 371011 Immunisation and Vaccine Preventable Diseases Division UK Health Security Agency London UK; ^3^ Centre for Thrombosis and Haemostasis St Thomas' Hospital Guy’s and Saint Thomas’ NHS Foundation Trust London UK; ^4^ Oxford University Hospitals NHS Foundation Trust Oxford NIHR Biomedical Research Centre Oxford UK; ^5^ 6396 Radcliffe Department of Medicine University of Oxford Oxford UK; ^6^ Wolfson Institute of Population Health Queen Mary University of London London UK; ^7^ 9744 Barts Health NHS Trust London UK; ^8^ Department of Haematology Royal Berkshire Hospital NHS Foundation Trust London UK; ^9^ 4615 Patient Experience Research Centre School of Public Health Imperial College London London UK

**Keywords:** COVID‐19, COVID‐19 vaccines, SARS‐CoV‐2, thrombocytopenia, thrombosis

## Abstract

**Background:**

Several studies have found increased risks of thrombosis with thrombocytopenia syndrome (TTS) following the ChAdOx1 vaccination. However, case ascertainment is often incomplete in large electronic health record (EHR)‐based studies.

**Objectives:**

To assess for an association between clinically validated TTS and COVID‐19 vaccination.

**Methods:**

We used the self‐controlled case series method to assess the risks of clinically validated acute TTS after a first COVID‐19 vaccine dose (BNT162b2 or ChAdOx1) or severe acute respiratory syndrome coronavirus 2 (SARS‐CoV‐2) infection. Case ascertainment was performed uninformed of vaccination status via a retrospective clinical review of hospital EHR systems, including active ascertainment of thrombocytopenia.

**Results:**

One hundred seventy individuals were admitted to the hospital for a TTS event at the study sites between January 1 and March 31, 2021. A significant increased risk (relative incidence [RI], 5.67; 95% confidence interval [CI], 1.02‐31.38) of TTS 4 to 27 days after ChAdOx1 was observed in the youngest age group (18‐ to 39‐year‐olds). No other period had a significant increase, although for ChAdOx1 for all ages combined the RI was >1 in the 4‐ to 27‐ and 28‐ to 41‐day periods (RI, 1.52; 95% CI, 0.88‐2.63; and (RI, 1.70; 95% CI, 0.73‐3.8, respectively). There was no significant increased risk of TTS after BNT162b2 in any period. Increased risks of TTS following a positive SARS‐CoV‐2 test occurred across all age groups and exposure periods.

**Conclusions:**

We demonstrate an increased risk of TTS in the 4 to 27 days following COVID‐19 vaccination, particularly for ChAdOx1. These risks were lower than following SARS‐CoV‐2 infection. An alternative vaccine may be preferable in younger age groups in whom the risk of postvaccine TTS is greatest.

**Essentials**

- Thrombosis with thrombocytopenia syndrome (TSS) may be induced by the COVID‐19 vaccination.
- We used a retrospective clinical audit (complete case ascertainment) to assess this association.
- There is an increased risk of TTS in the short‐term (4‐27 days) following COVID‐19 vaccination.
- The association is stronger in younger people vaccinated with the Oxford/AstraZeneca vaccine.

## 1 BACKGROUND AND RATIONALE

Early clinical trials did not detect any major adverse effects from either of the first two COVID‐19 vaccines used in the UK COVID‐19 immunization program: ChAdOx1 nCov‐19 (Oxford/AstraZeneca, Cambridge, England) and BNT162b2 mRNA (Pfizer/BioNTech, New York, NY, USA).^1^, ^2^

However, by March 10, 2021, the European Medicines Agency reported that 30 cases of thromboembolic events, which were predominantly venous, had been flagged among over 5 million ChAdOx1 recipients in Europe.^3^ Subsequent reports defined an unusual postvaccination thrombosis with thrombocytopenia syndrome (TTS)^4^ in multiple countries.^5^, ^6^, ^7^, ^8^ These reports have described a particular combination of thrombosis in unusual sites and thrombocytopenia occurring predominantly within 5 to 30 days after vaccination. This combination of features has become widely known as vaccine‐induced immune thrombocytopenia and thrombosis (VITT).^9^ The United Kingdom’s Medicines & Healthcare products and Regulatory Agency (MHRA) had received, as of September 29, 2021, 421 reports of major thromboembolic events with concurrent thrombocytopenia through its Yellow Card Scheme, after 41 million first doses and 38 million second doses of the ChAdOx1 vaccine.^5^ The overall incidence after first or unknown doses was 15.1 per million doses.^5^ The MHRA Yellow Card Scheme estimates derive from voluntary reporting, meaning cases may be misclassified as vaccine associated by the clinician based on guidelines and publicity at the time regarding any possible association. Indeed, the published UK experience of VITT recognizes 220 definite and probable cases over a similar time period.^10^ However, multiple large population‐based analyses have found increased risk of hematologic and vascular events that led to hospital admission or death in the weeks following first doses of the ChAdOx1 nCoV‐19 in the United Kingdom^11^, ^12^, ^13^ and Catalonia,^14^ and higher than expected rates of cerebral venous sinus thrombosis (CVST) in the ChAdOx1‐vaccinated population in Denmark and Norway.^3^

These studies mainly used electronic health record (EHR) systems and therefore benefitted from higher power; however, their case ascertainment was reliant on the accuracy of coding of hospital diagnoses. This can be incomplete, as reported in a recent Scottish study that demonstrated that ascertainment of CVST from diagnostic codes on hospital discharge records had low sensitivity.^15^

In this study, we investigate whether there is an increased risk of TTS following COVID‐19 vaccination based on a clinical audit of hospital radiology reports and clinical case notes across four hospital sites in England. This approach therefore allows for more complete case ascertainment than previous studies.

## 2 METHODS

### 2.1 Overview

We used the self‐controlled case series (SCCS) method to assess the short‐term risks of clinically validated acute TTS after a first COVID‐19 vaccine dose (BNT162b2 or ChAdOx1, primary exposures) or severe acute respiratory syndrome coronavirus 2 (SARS‐CoV‐2) infection (polymerase chain reaction–positive test, secondary exposure). Case ascertainment was performed manually by clinical hematologists uninformed of vaccination status via a retrospective clinical review of hospital EHR systems across four hospital sites in England.

### 2.2 Case identification

**FIGURE 1:**
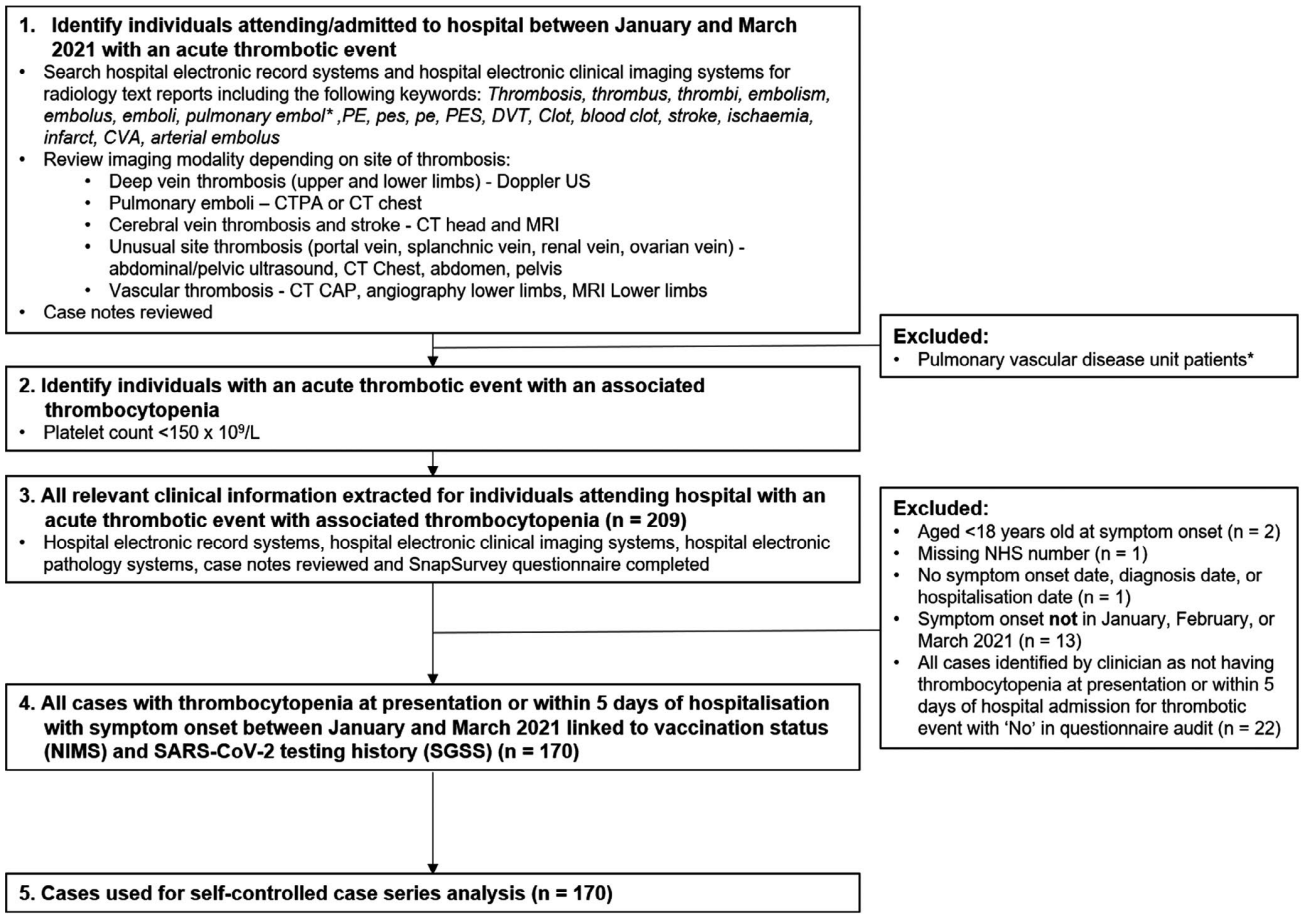
Flow diagram outlining case ascertainment and cohort refinement. *Patients in the pulmonary vascular disease unit excluded due to not being acute (all under follow‐up or long‐term care). CT, computed tomography; CT CAP, computed tomography of the chest, abdomen, and pelvis; CTPA, computed tomography pulmonary angiography; CVA, cerebrovascular accident; DVT, deep vein thrombosis; NIMS, National Immunisation Management Service; PE, pulmonary embolism; SARS‐CoV‐2, severe acute respiratory syndrome coronavirus 2; SGSS, Second Generation Surveillance System

All adults (aged >18 years) with an acute thrombotic event (venous or arterial thromboembolism) associated with a new‐onset thrombocytopenia between January 1 and March 31, 2021, at four hospitals in England were identified. The case ascertainment process is outlined in Figure 1. Platelet counts were actively sought from all identified thrombosis cases to determine the specific syndrome of TTS among the most common occurrence of isolated thrombosis. We defined TTS as an acute thrombotic event (venous or arterial thrombosis) associated with a new‐onset thrombocytopenia (platelet count <150 × 10^9^/L at presentation or within 5 days of hospital admission for a thrombotic event). We included all people aged ≥18 years at the time of the TTS event. We excluded any cases with symptom onset outside of the study period (January 1 to March 31, 2021), and any cases identified by the clinician as not having thrombocytopenia at presentation or within 5 days of hospital admission for the thrombotic event.

All identified cases were then linked to their vaccination status and SARS‐CoV‐2 testing history via the National Immunisation Management Service and Second Generation Surveillance System databases (Figure 1). We constructed the vaccination status (unvaccinated, first or second dose of ChAdOx1, or first or second dose of BNT162b2) of the population at date of symptom onset. If a case tested positive for SARS‐CoV‐2 on multiple dates, then the date in the 4‐ to 41‐day period before symptom onset was used as the primary exposure date.

### 2.3 Statistical methods

The SCCS method^16^ was used to test the hypothesis of an increased risk of TTS in four risk periods of 4 to 13, 14 to 27, 28 to 41, and 4 to 27 days after COVID‐19 vaccination and SARS‐CoV‐2–positive test, where day 0 is the day of vaccination or SARS‐CoV‐2 positive test, and day 1 the following calendar day, and so on. These risk periods were defined a priori based on early case reports of thrombosis with thrombocytopenia syndrome occurring predominantly within 5 to 30 days after vaccination.^4^ In the SCCS method, cases act as their own controls as the incidence of the event in predefined risk periods following vaccination is compared to the incidence outside the risk period, generating a relative incidence (RI) measure automatically controlled for time‐invariant confounding.^16^ We investigated the risk associated with first COVID‐19 vaccine dose (any manufacturer), first dose of ChAdOx1, and first dose of BNT162b2. We used the standard SCCS approach, with adjustment for 15‐day intervals from January 1, 2021, to account for temporal changes in background rates. Models were fitted using a conditional Poisson regression, with resultant estimates representing the RI of hospital admission due to TTS or a SARS‐CoV‐2–positive test relative to baseline periods and their 95% confidence intervals (CIs). Exposure terms for both vaccines and for infection with SARS‐CoV‐2 were included in all models. The observation period (person‐time) for each individual was from January 1 to March 31, 2021. We performed the 4‐ to 27‐day period analysis for the vaccination exposures with the 28‐day prevaccination low period included as a sensitivity analysis to assess whether experiencing a TTS event increases the chance of subsequently being vaccinated. We also performed the 4‐ to 27‐day period analysis with follow‐up starting on day of vaccination and ending on the earliest of date of second vaccination dose; March 31, 2021; or 90 days after the first dose. We investigated the associations in three age‐stratified subgroups: 18 to 39, 40 to 64, and 65+ years. The effect (RI) size is the main outcome of our study. In reporting and interpreting our results, we present both the substantive significance (effect size) and statistical significance (*P* ≤ .05). Effect size helps understand the magnitude of differences found, whereas statistical significance examines whether the findings are likely to be due to chance. Both are essential to understand the full impact of our findings. Analysis was carried out using Stata version 15 (StataCorp, College Station, TX, USA) and R version 4.04 (R Foundation for Statistical Computing, Vienna, Austria).

## 3 RESULTS

### 3.1 Descriptive statistics and characteristics

Table 1 outlines the characteristics of 170 individuals identified as being admitted to the hospital for a TTS event at one of the study sites with symptom onset (or hospitalization) between January 1 and March 31, 2021. Most cases (61.4%) were aged >65. Overall, 99 individuals (58.2%) were not vaccinated at the time of their symptom onset, 68 (40.0%) had received a first dose, and 3 (1.76%) had received a second dose. Among the vaccinated cases, 32 people (45.0%) had received BNT162b2 and 39 (54.9%) had received ChAdOx1. Only 8 individuals (4.71%) had a PF4 ELISA result, 3 of which were negative and 5 positive.

**TABLE 1:** Baseline characteristics of 170 individuals who had an acute arterial or venous thrombotic event associated with thrombocytopenia (platelet count <150 x109/L) at the study hospital centers between January and March 2021, stratified by COVID‐19 vaccination status at time of symptom onset

Variable	Total	Not vaccinated^b^	Any COVID‐19 vaccine		ChAdOx1 nCoV‐19 (Oxford/ AstraZeneca)	BNT162b2mRNA (Pfizer/BioNTech)
			One dose	Two doses		
Age at symptom onset, y						
Median	66	64	74	88	67	81
18‐39, n (%)	16 (9.5)	10 (62.5)	6 (37.5)	0 (0.0)	6 (37.5)	0 (0.0)
40‐64, n (%)	63 (37.1)	46 (73.0)	17 (27.0)	0 (0.0)	13 (20.6)	4 (6.3)
65+, n (%)	91 (53.5)	43 (47.3)	45 (50.6)	3 (3.4)	20 (22.5)	28 (31.5)
Sex, n (%)						
Female	56 (32.9)	33 (58.9)	22 (39.3)	1 (1.8)	14 (25.0)	9 (16.1)
Male	114 (67.1)	66 (57.9)	46 (40.4)	2 (1.8)	25 (21.9)	23 (20.2)
Ethnicity, n (%)						
White	89 (53.3)	47 (52.8)	40 (44.9)	2 (2.2)	21 (23.6)	21 (23.6)
Mixed	3 (1.8)	3 (100.0)	0 (0.0)	0 (0.0)	0 (0.0)	0 (0.0)
Asian/ Asian British	12 (7.2)	11 (91.7)	1 (8.3)	0 (0.0)	1 (8.3)	0 (0.0)
Black/African/Caribbean/Black British	12 (7.2)	10 (83.3)	2 (16.7)	0 (0.0)	2 (16.7)	0 (0.0)
Other ethnic group	1 (0.6)	1 (100.0)	0 (0.0)	0 (0.0)	0 (0.0)	0 (0.0)
Unknown	50 (29.9)	27 (54.0)	22 (44.0)	1 (2.0)	12 (24.0)	11 (22.0)
Body mass index, n (%)						
Underweight (<18.5)	11 (6.5)	6 (54.5)	5 (45.5)	0 (0.0)	1 (9.1)	4 (36.4)
Normal weight (18.5‐24.9)	48 (28.2)	23 (47.9)	25 (52.1)	0 (0.0)	13 (27.1)	12 (25.0)
Overweight (25‐29.9)	33 (19.4)	23 (69.7)	10 (30.3)	0 (0.0)	7 (21.2)	3 (9.1)
Obesity (>30)	45 (26.5)	26 (57.8)	17 (37.8)	2 (4.4)	14 (31.1)	5 (11.1)
Not known	33 (19.4)	21 (63.6)	11 (33.3)	1 (3.0)	4 (12.1)	8 (24.2)
Clinically extremely vulnerable,^a^ n (%)						
No	119 (70.0)	72 (60.5)	46 (38.7)	1 (0.8)	25 (21.0)	22 (18.5)
Yes	51 (30.0)	27 (52.9)	22 (43.1)	2 (3.9)	14 (27.5)	10 (19.6)
Care home resident, n (%)						
No	169 (99.4)	99 (58.6)	67 (39.6)	3 (1.8)	38 (22.5)	32 (18.9)
Yes	1 (0.6)	0 (0.0)	1 (100.0)	0 (0.0)	1 (100.0)	0 (0.0)
Thrombotic event, n (%)						
Venous	88 (51.8)	64 (72.7)	23 (26.1)	1 (1.1)	11 (12.5)	13 (14.8)
Arterial	28 (16.5)	12 (42.9)	14 (50.0)	2 (7.1)	8 (28.6)	8 (28.6)
Both	7 (4.1)	2 (28.6)	5 (71.4)	0 (0.0)	3 (42.9)	2 (28.6)
Unknown	47 (27.7)	21 (44.7)	26 (55.3)	0 (0.0)	17 (36.2)	9 (19.1)
Month of symptom onset date,^c^ n (%)						
Jan 2021	62 (36.5)	52 (83.9)	10 (16.1)	0 (0.0)	1 (1.6)	9 (14.5)
Feb 2021	58 (34.1)	37 (63.8)	19 (32.8)	2 (3.4)	12 (20.7)	9 (15.5)
Mar 2021	50 (29.4)	10 (20.0)	39 (78.0)	1 (2.0)	26 (52.0)	14 (28.0)
Smoking status, n (%)						
Smoker	31 (18.2)	19 (61.3)	12 (38.7)	0 (0.0)	9 (29.0)	3 (9.7)
Nonsmoker	52 (30.6)	33 (63.5)	18 (34.6)	1 (1.9)	7 (13.5)	12 (23.1)
Unknown	87 (51.2)	47 (54.0)	38 (43.7)	2 (2.3)	23 (26.4)	17 (19.5)
Pregnancy status, n (%)						
Pregnant	1 (0.6)	1 (100.0)	0 (0.0)	0 (0.0)	0 (0.0)	0 (0.0)
Not pregnant	165 (97.1)	96 (58.2)	66 (40.0)	3 (1.8)	37 (22.4)	32 (19.4)
Unknown	4 (2.4)	2 (50.0)	2 (50.0)	0 (0.0)	2 (50.0)	0 (0.0)
Underlying comorbities, n (%)						
>3	41 (24.1)	28 (68.3)	13 (31.7)	0 (0.0)	9 (22.0)	4 (9.8)
2	55 (32.4)	30 (54.5)	24 (43.6)	1 (1.8)	14 (25.5)	11 (20.0)
1	29 (17.1)	19 (65.5)	10 (34.5)	0 (0.0)	6 (20.7)	4 (13.8)
None	45 (26.5)	22 (48.9)	21 (46.7)	2 (4.4)	10 (22.2)	13 (28.9)
Death within 28 days of event onset, n (%)						
No	137 (80.1)	78 (56.9)	56 (40.9)	3 (21.9)	33 (24.1)	26 (19.0)
Yes	33 (19.4)	21 (63.6)	12 (36.4)	0 (0.00)	6 (18.2)	6 (18.2)
COVID‐19 infection, n (%)						
No	144 (84.7)	80 (55.6)	61 (42.4)	3 (2.1)	37 (25.7)	27 (18.8)
Yes: 0‐27 days before event onset	26 (15.3)	19 (73.1)	7 (26.9)	0 (0.0)	2 (7.7)	5 (19.2)
Yes: >27 days before event onset	0 (0.0)	0 (0.00)	0 (0.00)	0 (0.00)	0 (0.00)	0 (0.00)
Vaccination (no. of days prior to symptom onset date) , n (%)						
No	121 (71.2)	99 (81.8)	19 (15.7)	3 (2.5)	6 (5.0)	16 (13.2)
Dose 1: 0‐3	5 (2.9)	0 (0.0)	5 (100.0)	0 (0.0)	4 (80.0)	1 (20.0)
Dose 1: 4‐13	17 (10.0)	0 (0.0)	17 (100.0)	0 (0.0)	11 (64.7)	6 (35.3)
Dose 1: 14‐27	13 (7.6)	0 (0.0)	13 (100.0)	0 (0.0)	10 (76.9)	3 (23.1)
Dose 1: 28‐41	14 (8.2)	0 (0.0)	14 (100.0)	0 (0.0)	8 (57.1)	6 (42.9)
Dose 2: 0‐27	0 (0.0)	0 (0.00)	0 (0.00)	0 (0.00)	0 (0.00)	0 (0.00)
Cause of thrombocytopenia, n (%)						
Liver cirrhosis	7 (4.1)	5 (71.4)	2 (28.6)	0 (0.0)	1 (14.3)	1 (14.3)
Antiphospholipid syndrome	0 (0.0)	0 (0.00)	0 (0.00)	0 (0.00)	0 (0.00)	0 (0.00)
HIV	0 (0.0)	0 (0.00)	0 (0.00)	0 (0.00)	0 (0.00)	0 (0.00)
SLE	0 (0.0)	0 (0.00)	0 (0.00)	0 (0.00)	0 (0.00)	0 (0.00)
Drug‐induced	14 (8.2)	8 (57.1)	6 (42.9)	0 (0.0)	3 (21.4)	3 (21.4)
DIC	4 (2.4)	1 (25.0)	3 (75.0)	0 (0.0)	1 (25.0)	2 (50.0)
TTP	0 (0.0)	0 (0.00)	0 (0.00)	0 (0.00)	0 (0.00)	0 (0.00)
Other	54 (31.8)	41 (75.9)	12 (22.2)	1 (1.9)	6 (11.1)	7 (13.0)
Unknown	91 (53.5)	44 (48.4)	45 (49.5)	2 (2.2)	28 (30.8)	19 (20.9)
Site of thrombosis, n (%)						
Cerebral vein	5 (2.9)	2 (40.0)	3 (60.0)	0 (0.0)	3 (60.0)	0 (0.0)
Splanchnic (including hepatic portal)	8 (4.7)	4 (50.0)	3 (37.5)	1 (12.5)	3 (37.5)	1 (12.5)
Pulmonary embolism	88 (51.8)	54 (61.4)	33 (37.5)	1 (1.1)	18 (20.5)	16 (18.2)
Deep vein thrombosis	39 (22.9)	25 (64.1)	14 (35.9)	0 (0.0)	8 (20.5)	6 (15.4)
Arterial	39 (22.9)	16 (41.0)	22 (56.4)	1 (2.6)	13 (33.3)	10 (25.6)
Other	19 (11.2)	10 (52.6)	9 (47.4)	0 (0.0)	6 (31.6)	3 (15.8)
None	1 (0.6)	0 (0.0)	1 (100.0)	0 (0.0)	1 (100.0)	0 (0.0)

Note

Percentages are column percentages in the first column and row totals in all other columns unless stated otherwise.

Abbreviations: DIC, disseminated intravascular coagulation; SLE, systemic lupus erythematosus; TTP, thrombotic thrombocytopenic purpura.

^a^ UK government definition of clinically extremely vulnerable available fromhttps://www.nidirect.gov.uk/articles/coronavirus‐covid‐19‐definitions‐clinically‐extremely‐vulnerable‐and‐vulnerable

^b^ People never vaccinated or vaccinated after onset of thrombotic event.

^c^ Thrombosis admission date used as proxy for symptom onset date when unavailable (n = 1). If symptom onset date and admission date were unavailable, thrombosis diagnosis date was used as a proxy (n = 0). Cases with missing data for all three of these dates were excluded (n = 1).

### 3.2 Relative incidence estimates – primary exposures (first dose of any COVID‐19 vaccine, BNT162b2, and ChAdOx1)

Overall, 17 TTS cases occurred 4 to 13 days after vaccination with any COVID‐19 vaccine, 13 occurred 14 to 27 days after vaccination, and 14 occurred 28 to 41 days after vaccination (Table 2). A larger proportion of these admissions were following ChAdOx1 (64.7% [11/17] 4‐13 days after vaccination; 76.9% [10/13] 14‐27 days after vaccination; and 57.1% [8/14] 28‐41 days after vaccination) than following BNT162b2. Most TTS symptoms (74.1%; 126/170) did not start within the 4‐ to 41‐day postvaccination risk period of interest.

**TABLE 2:** RIs and 95% CIs for an acute thrombotic event with thrombocytopenia in different time periods after COVID‐19 vaccination and testing positive for SARS‐CoV‐2 using self‐controlled case series analysis

Period of risk after vaccination or positive test, d^c^	Any vaccine (first dose)		BNT162b2 mRNA (Pfizer/BioNTech, first dose)		ChAdOx1 nCoV‐19 (Oxford/AstraZeneca, first dose)		SARS‐CoV‐2–positive test	
	RI (95% CI)	Number of events	RI (95% CI)	Number of events	RI (95% CI)	Number of events	RI (95% CI)	Number of events
All ages								
Baseline^a^	1.00	126	1.00	155	1.00	141	1.00	154
4‐13	1.65 (0.95‐2.88)	17	1.46 (0.58‐3.70)	6	1.83 (0.91‐3.69)	11	5.61 (2.31‐13.64)	11
14‐27	0.89 (0.48‐1.65)	13	0.38 (0.11‐1.28)	3	1.43 (0.68‐3.03)	10	1.88 (0.63‐5.61)	5
28‐41	1.29 (0.69‐2.41)	14	0.97 (0.38‐2.46)	6	1.70 (0.73‐3.98)	8	N/A	0
4‐27	1.21 (0.78‐1.87)	30	0.82 (0.38‐1.75)	9	1.52 (0.88‐2.63)	21	4.36 (1.95‐9.73)	16
4‐27^b^	1.63 (0.32‐8.43)	30	0.68 (0.31‐1.48)	9	1.51 (0.82‐2.78)	21	N/A	16
4‐27^d^	1.00 (0.45‐2.22)	30	0.86 (0.30‐2.51)	9	1.08 (0.45‐2.57)	21	N/A	16
18‐ to 39‐year‐olds								
Baseline^a^	1.00	11	1.00	16	1.00	11	1.00	16
4‐13	5.61 (0.92‐34.35)	3	N/A	0	5.61 (0.92‐34.35)	3	N/A	0
14‐27	3.45 (0.39‐30.61)	2	N/A	0	3.45 (0.39‐30.61)	2	N/A	0
28‐41	N/A	0	N/A	0	N/A	0	N/A	0
4‐27	5.67 (1.02‐31.38)	5	N/A	0	5.67 (1.02‐31.38)	5	N/A	0
4‐27^b^	9.00 (0.74‐109.03)	5	N/A	0	9.00 (0.74‐109.03)	5	N/A	0
40‐ to 64‐year‐olds								
Baseline^a^	1.00	52	1.00	60	1.00	54	1.00	55
4‐13	1.59 (0.60‐4.18)	6	1.44 (0.26‐8.03)	2	1.60 (0.51‐5.05)	4	8.45 (1.89‐37.80)	5
14‐27	0.60 (0.16‐2.15)	3	N/A	0	0.99 (0.26‐3.71)	3	4.14 (0.79‐21.76)	3
28‐41	0.78 (0.17‐3.73)	2	0.85 (0.09‐7.62)	1	0.74 (0.09‐6.20)	1	N/A	0
4‐27	1.09 (0.47‐2.53)	9	0.59 (0.11‐3.25)	2	1.35 (0.53‐3.49)	7	7.92 (2.01‐31.26)	8
4‐27^b^	N/A	9	0.68 (0.13‐3.66)	2	1.74 (0.58‐5.16)	7	N/A	8
≥65‐year‐olds								
Baseline^a^	1.00	61	1.00	77	1.00	73	1.00	81
4‐13	1.26 (0.56‐2.83)	8	1.20 (0.38‐3.82)	4	1.24 (0.39‐3.93)	4	4.68 (1.48‐14.82)	6
14‐27	0.76 (0.34‐1.71)	8	0.41 (0.11‐1.49)	3	1.28 (0.43‐3.80)	5	0.99 (0.20‐4.87)	2
28‐41	1.47 (0.72‐2.96)	12	0.90 (0.31‐2.59)	5	2.49 (0.91‐6.81)	7	N/A	0
4‐27	0.94 (0.52‐1.69)	16	0.80 (0.33‐1.92)	7	1.09 (0.48‐2.48)	9	3.12 (1.11‐8.78)	8
4‐27^b^	0.79 (0.14‐4.39)	16	0.48 (0.19‐1.22)	7	0.76 (0.31‐1.89)	9	N/A	8

N/A (not applicable); SARS‐CoV‐2, severe acute respiratory syndrome coronavirus 2. Analysis not applicable due to there being no events of interest in the specified time frame for the cohort.

^a^ Outside 4‐ to 41‐day exposure risk period.

^b^ With 28‐day prevaccination low period.

^c^ Day 0 is the day of vaccination or SARS‐CoV‐2–positive test.

^d^ Follow‐up started on day of vaccination and ended on the earliest of date of second vaccination dose; March 31, 2021; or 90 days after first dose.

A nonsignificant increased risk of TTS was observed in the 4‐ to 13‐day period after COVID‐19 vaccination (RI, 1.65; 95% CI, 0.95‐2.88). This risk attenuated when investigating the larger 4‐ to 27‐day postexposure period (RI, 1.21; 95% CI, 0.78–1.87). A small and nonsignificant increased risk of TTS following BNT162b2 was observed only in the 4‐ to 13‐day period after vaccination (RI, 1.46; 95% CI, 0.58–3.70), and there was an opposite direction of effect (decreased RI of TTS) for all other periods included in the analysis. This contrasts the periods following exposure to ChAdOx1, with all exposure windows studied having RI point estimates above 1 (ranging [for all ages] from 1.08; 95% CI, 0.45‐2.57 [4‐ to 27‐day period] to 1.83; 95% CI, 0.91‐3.69 4‐ to 13‐day period]). Results were similar for the sensitivity analysis with the 28‐day prevaccination low period included; however, inclusion of the 0 to 90 days following the first dose in follow‐up attenuated the effect for the 4 to 27 days following ChAdOx1 (RI, 1.08; 95% CI, 0.45‐2.57).

Larger increased risks were observed following ChAdOx1 in the youngest age group (18‐ to 39‐year‐olds). None of this age group had received BNT162b2. An increased risk (RI, 5.67; 95% CI, 1.02‐31.38) of TTS 4 to 27 days following ChAdOx1 was observed. The direction and size of risks were mixed in the 40‐ to 64‐year‐old and ≥65‐year‐old groups, and all had wide 95% CIs due to low sample size. Across all age groups, an increased but nonsignificant risk was observed for the 4‐ to 13‐day period after vaccination with any COVID‐19 vaccine.

### 3.3 Relative incidence estimates – secondary exposure (SARS‐CoV‐2–positive test)

Sixteen cases tested positive for SARS‐CoV‐2 in the 4 to 41 days before their TTS symptom onset. Increased risks of TTS following a positive SARS‐CoV‐2 test were observed across all age groups and exposure periods, except in the 18‐ to 39‐year‐olds, where no cases tested positive in the 4‐ to 41‐day exposure window. An almost 5‐fold increase in TTS risk was observed in the 4 to 27 days following a positive test (RI, 4.36; 95% CI, 1.95–9.73), which increased to 5.61 (95% CI, 2.31‐13.64) when examining the smaller 4‐ to 13‐day exposure window.

## 4 DISCUSSION

Our study provides evidence of an increased risk of TTS 4 to 27 days after a first dose of ChAdOx1 in those aged 18 to 39 years old. In other age groups, point estimates were also >1 for ChadOx1 and any COVID‐19 vaccine but were not statistically significant. No increased risk following the first dose of BNT162b2 was observed. Overall, risks were multiple‐fold higher in the exposure periods following a positive SARS‐CoV‐2 test than COVID‐19 vaccination.

To our knowledge, this is the first study to use clinician‐verified validation of TTS cases for cohort assembly to then investigate a potential association with COVID‐19 vaccination. This labor‐intensive form of data collection enabled complete, robust case ascertainment in the source population. Importantly, platelet counts were ascertained for all thrombotic events, to identify the specific syndrome of TTS among the more common occurrence of isolated thrombosis. Once linked to vaccination data, this also enabled clinician follow‐up of patients with suspected VITT. Unlike other studies, our study does not rely on adverse event reporting or potentially inaccurate diagnostic coding of hospital discharge records, reducing misclassification bias. Clinicians performing the data collection were not directly informed of vaccination and SARS‐CoV‐2 infection status unless included in case notes. Given that during the study period there was very limited awareness of VITT among clinicians (the syndrome was not recognized widely by UK hematologists until March 2021), vaccination was unlikely to have been routinely recorded for a patient presenting with a thrombotic event. This reduced the likelihood of any reporting bias and limited clinical detection bias in the hospital records used. Linkage to national, validated databases of vaccination and SARS‐CoV‐2 testing dates means that the exposure time periods under study here are unlikely to be under‐ or overestimations.

Our use of the SCCS method overcomes many of the commonly reported issues with cohort or case‐control studies. Use of either of these observational study methods within the context of a rapidly progressing pandemic, including the emergence of the alpha variant at the end of 2020 and a large‐scale vaccination drive, would make matching of cases and controls difficult without introducing significant confounding. Unvaccinated controls or cases may retain this status for only short periods of time. At the beginning of the study period, only care home residents, frontline health and social care workers, and individuals aged ≥80 years were eligible for COVID‐19 vaccination in England, whereas by the end of the period this group included those aged 16 to 64 with underlying health conditions and all individuals aged ≥50.^17^ Our study design uses within‐person comparison, therefore reducing confounding for all fixed characteristics, while temporal confounding was controlled for using 15‐day period adjustment.

The main limitation of our study is a lack of power—the time‐intensive, manual nature of the data collection performed here meant that the geographical coverage and time frame for the study was small. Therefore, although the effect sizes provide evidence of TTS following ChAdOx1 vaccination, many of the RI point estimates are not statistically significant. We were also unable to convert the RIs into rates or estimates of attributable risk due to the lack of accurate data on the catchment populations of the hospital study sites; many are tertiary referral centers, and we therefore could not calculate a denominator. A recent UK‐based study estimated the attributable risk of CVST per 1 million first COVID‐19 doses as 16.1 (95% CI, 15.0‐17.7) in 15‐ to 39‐year‐olds versus 3.2 (95% CI, 1.7‐4.0) in 40‐ to 64‐year‐olds,^18^ highlighting the large difference in absolute risk between these age groups; generation of a similar absolute measure in our study would aid contextualization of the RIs. Inclusion of the 0 to 90 days following the first dose in the follow‐up period attenuated after ChAdOx1 RI; this analysis has the advantage of not being affected by the event itself affecting subsequent vaccination (eg, due to death), but the disadvantage of having less power due to the limited control window. This demonstrates that there is some potential for bias when using the prevaccination person‐time, and this is a study limitation. This study may underestimate rates of TTS in older people because our analysis is based on hospital admissions; fragile older people with symptoms of possible thrombosis, such as headache, after vaccination may not have necessarily been referred into a hospital before the syndrome of VITT was recognized.

Our results broadly echo those from similar studies.^11^, ^12^ A Scottish study of EHR data found that nested case‐control effect sizes were larger than those estimated in the same cohort using SCCS, likely due to the presence of residual confounding or confounding by indication in the case‐control study.^11^ They detected an increased risk (relative risk [RR], 1.98; 95% CI, 1.29‐3.02) of thrombocytopenia 0 to 28 days following ChAdOx1, but not for arterial thromboembolic events (RR, 0.97; 95% CI, 0.83‐0.11).^11^ Similar results were reported in an English SCCS analysis of risk of thrombocytopenia (incidence rate ratio [IRR], 1.33; 95% CI, 1.19–1.47) and venous thromboembolism (IRR, 1.10; 95% CI, 1.02‐1.18)^8^, ^9^, ^10^, ^11^, ^12^, ^13^, ^14^ days after ChAdOx1 vaccination.^12^ The same study also found the risks of hematologic and vascular events to be substantially higher after SARS‐CoV‐2 infection than after vaccination.^12^ These studies benefitted from higher power but are not directly comparable to ours due to using existing hospitalization discharge codes for outcome assignment, unlike our manual classification of cases as TTS.

In conclusion, we found some evidence of an increased risk of TTS in the time period 4 to 27 days following COVID‐19 vaccination, particularly for ChAdOx1. However, the risk was much lower than following SARS‐CoV‐2 infection. Our findings suggest that in younger people, an alternative vaccine may be preferable and short‐term monitoring of symptoms 4 to 27 days after vaccination are necessary. This supports the UK Joint Committee on Vaccine and Immunisation recommendation to preferentially use mRNA vaccines in people <40 years old given the ChAdOx1 risk‐benefit profile in younger age groups. Similar studies using robust clinical case ascertainment of TTS over larger time frames and geographies are required to confirm our findings.

## RELATIONSHIP DISCLOSURE

All authors declare no relevant conflicts of interest.

## AUTHOR CONTRIBUTIONS

HH performed data cleaning, interpretation, and analysis, and wrote the report. CJA and BJH conceptualized and designed the study, helped in data interpretation, contributed to data collection, and helped refine and draft the report for important intellectual content. NA and JS contributed to the design of the study, analysis, and interpretation of the data and revised the report critically for important intellectual content. GA and MR contributed to the conceptualization and design of the study and revised the report critically for important intellectual content. GB, AH, KB, MD, DK, HL, CS, EH, SS, CL, JD, PKM, SB, VM, NMAE, and SB performed the data collection for the report. All authors approved the final version of this article for publication.
